# First Insights on Organic Cosolvent Effects on FhuA Wildtype and FhuA Δ1-159

**DOI:** 10.3390/ijms13022459

**Published:** 2012-02-22

**Authors:** Stefanie-Joana Tenne, Ulrich Schwaneberg

**Affiliations:** Lehrstuhl für Biotechnologie, RWTH Aachen University, Worringer Weg 1, Aachen, D-52074, Germany; E-Mail: j.tenne@biotec.rwth-aachen.de

**Keywords:** secondary structure, circular dichroism, membrane protein, organic cosolvent, β-barrel, FhuA, precipitation, structural integrity

## Abstract

Circular dichroism (CD) and deconvolution were used to study the structural integrity of a “plugged” and an “open” FhuA transmembrane channel protein in the presence of varied concentrations of tetrahydrofuran (THF), ethanol (EtOH) and chloroform/methanol (C/M). FhuA is an *Escherichia coli* outer membrane protein (78.9 kDa) consisting of 22 β-sheets and an internal globular cork domain which acts as an iron transporter. FhuA and the deletion variant FhuA Δ1-159 showed comparable and remarkable resistance in the presence of THF (≤40 vol%) and EtOH (≤10 vol%). In C/M, significant differences in structural resistance were observed (FhuA stable ≤10 vol%; FhuA Δ1-159 ≤1 vol%). Deconvolution of CD-spectra for FhuA and FhuA Δ1-159 yielded β-sheet contents of 61 % (FhuA) and 58% (FhuA Δ1-159). Interestingly, FhuA and FhuA Δ1-159 had comparable β-sheet contents in the presence and absence of all three organic cosolvents. Additionally, precipitated FhuA and FhuA Δ1-159 (in 40 vol% C/M or 65 vol% THF) redissolved by supplementing the detergent *n*-octyl-oligo-oxyethylene (oPOE).

## 1. Introduction

Forces controlling the overall structure of proteins include electrostatic interactions, van der Waals forces, disulfide bridges and π-π stacking of aromatic site chains [1–3]. Membrane proteins have three regions that interact with the surrounding environment: (i) regions interacting with water; (ii) regions in direct contact with the lipid membrane; and (iii) regions facing the protein interior [2]. Membrane proteins fulfill a variety of complex functions as diffusion pores, substrate specific transporters, membrane anchors, signal transduction and compound conversion [4]. Antiparallel β-sheets of integral membrane proteins often show a meander topology, which can form a β-barrel structure and enable translocation of molecules through biological membranes. Hydrogen bonds are the main forces holding β-sheets together in a β-barrel protein. A common feature of β-barrel proteins is their frequently-discovered high resistance to heat, chaotropic salts, detergents and proteolysis [5]. The latter properties make β-barrel proteins attractive for applications in biocatalysis (hybrid catalysts), biotechnology (selective product recovery [6,7]), as well as medical applications through triggered compound release (e.g., pH [8], reduction [9] and light trigger [10]).

Gaining first insights on cosolvent effects that govern the structural integrity and solubility of β-barrel proteins are therefore important prerequisites for developing successfully hybrid catalysts, selective membranes and triggerable release systems. A “systematic” study for examining the structural integrity of an integral β-barrel membrane protein in the presence of different concentrations of organic solvents by CD^1^ has not, to our best knowledge, been reported.

In the current report, the organic cosolvents tetrahydrofuran (THF) [11], ethanol (EtOH) [8] and chloroform/methanol (C/M) were selected to investigate their effects on the iron transporter FhuA (ferric hydroxamate uptake protein component A) [12]. THF and EtOH were used for developing FhuA-based triggered release systems [10,13], modulating compound fluxes [14] and in hybrid catalysts synthesis [15]. FhuA is a monomeric two-domain outer membrane protein of *E. coli* with a molecular weight of 78.9 kDa consisting of 22 antiparallel β-strands (*C*-terminal, residues 161 to 723) and a NH_2_-terminal cork domain (residues 1 to 160) [12,16]. FhuA has a height of 69 Å and an elliptical cross section of 39 to 46 Å [16]. FhuA transports iron in the form of siderophores into *E. coli*. Furthermore it serves as a receptor for a number of bacteriophages (T5, T1, Φ80) and antibiotics (colicin M, albomycin) [17]. Removal of the cork domain (FhuA Δ1-159) resulted in a passive diffusion channel [12–15].

Differential scanning calorimetry (DSC) [18,19], CD [20], FTIR and NMR [21] can be used for studying the structural integrity of proteins. FTIR was excluded due to a high signal-noise ratio of FhuA and its environment, composed of lipids and detergent. The size of FhuA (>60 kDa), possible multimer formation and the presence of large molar excess of detergent suggested that NMR should be excluded. DSC and CD were promising choices and CD finally selected. A DSC study on thermal resistance of FhuA wildtype, showing two transition centers, due to the presence of the cork domain, and FhuA Δ28-159, showing one transition centre, was published [5] (FhuA: *T*_m1_ = 64.0 °C and *T*_m2_ of 74.4 °C, FhuA Δ28-159: *T*_m_ = 61.6 °C).

In essence, we report a CD-based study on the structural integrity of FhuA and its mutant FhuA Δ1-159 in different concentrations of the organic solvents THF, EtOH and C/M.

## 2. Results and Discussion

In Section 2.1, the CD, as well as the deconvolution method, are described, including control experiments, in order to ensure that structural effects of cosolvents on FhuA and FhuA Δ1-159 can be determined by CD-spectra analysis. In Section 2.2, the obtained resistance results of FhuA and FhuA Δ1-159 in varied concentrations of THF, EtOH and C/M mixtures are reported and analyzed from a structural point of view. In Section 2.3, molecular reasons like water-stripping effect, dielectric constant and empirical logP concept are discussed for the cosolvents THF, EtOH and C/M.

In order to simplify the discussion, FhuA or FhuA Δ1-159, in the presence of oPOE detergent (1.05 vol%) in phosphate buffer (100 mM P*i*, pH 8, 1 mM EDTA), will be referred to as “standard” buffer in contrast to cosolvent supplemented buffers.

### 2.1. Results on CD-Measurements

**Figure 1:**
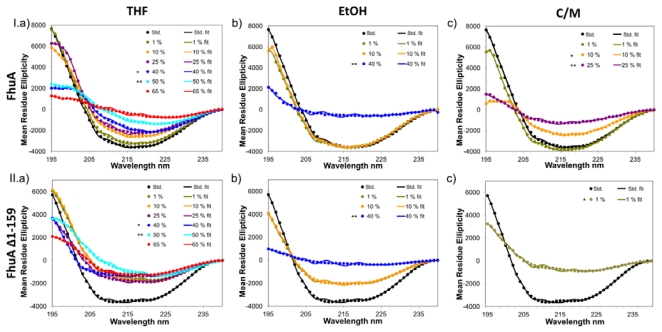
The respective amount of organic cosolvent (THF, EtOH or C/M) was added to 180 ng/μL of FhuA or FhuA Δ1-159 in a total volume of 150 μL. Samples were stirred for 75 min. CD-spectra were recorded using the Olis spectrapolarimeter model SDM 17, within a SUPRASIL cuvette with a pathlength of 0.5 mm. After subtraction of buffer baseline spectra and smoothing using a Savitzky-Golay smoothing filter, results were expressed in milli degrees. Data were converted into mean residue ellipticity and fitted using the CONTIN algorithm, implemented in the Dichroprot software. Original data (dotted lines) and fitted data (solid lines) of (**I**) FhuA and (**II**) FhuA Δ1-159 in (**a**) THF; (**b**) EtOH and (**c**) C/M are shown. Samples marked with one asterisk showed precipitation of FhuA or FhuA Δ1-159 upon addition of organic cosolvent which redissolved after incubation. Two asterisks indicate the FhuA or FhuA Δ1-159 precipitation events in which FhuA or FhuA Δ1-159 did not fully redissolve during CD-measurements.

Characteristic CD-spectra for a β-sheet peptide have a minimum of around 215 nm and a maximum around 195 nm. Peak variations are due to contributions of β-turns or antiparallel interactions between the different β-sheets [22]. Figure 1 shows the original and fitted CD-spectra of FhuA (I; upper three CD-spectra experiments) and FhuA Δ1-159 (II; lower three CD-spectra experiments) in “standard” buffer and in the presence of varied concentrations of the organic cosolvents THF (I.a/II.a), EtOH (I.b/II.b) and C/M (I.c/II.c). The CD-spectra of FhuA and FhuA Δ1-159 in a “standard” buffer show that FhuA and FhuA Δ1-159 are composed of a β-sheet structure. The recorded CD-spectra are nearly identical to a first recorded CD-spectrum of FhuA wildtype in 10 mM Tris (pH 7.2), 10 mM NaCl and 16.5 mM octyl glucoside buffer [23]. Comparison of FhuA to FhuA Δ1-159 in THF and EtOH show a similar trend and high resistance of the β-barrel structure towards the organic cosolvents. In both cases the maximum at around 195 nm decreases significantly in the presence of 40 vol% organic cosolvent. The single minimum at around 215 nm, which is typical for β-sheets, is decreased step-wise by the addition of cosolvent to the wildtype FhuA protein. In contrast to FhuA, addition of small amounts of THF or EtOH to FhuA Δ1-159 directly lowers the minima but the overall shape remains almost the same during the addition of any concentration of cosolvents. However, one has to be careful not to overinterpret differences in CD-spectra, for instance in the range of 205–240 nm (see Figure 1 IIa). As a general trend it was found that the resistance of the secondary structure of FhuA proteins towards C/M is much lower, compared to THF or EtOH (see Figure 1). In addition, the secondary structure of FhuA Δ1-159 is less resistant towards THF, EtOH and C/M, compared to the wildtype FhuA.

Supplementation of 40 vol% THF or 25 vol% EtOH (data not shown) during sample preparation of FhuA or FhuA Δ1-159 turned the protein solution from transparent to turbid. This phenomenon disappeared after incubation (75 min; see Figure 1, *). In the case of C/M, 10 vol% C/M (FhuA) or 1 vol% (FhuA Δ1-159) resulted in similar effects. Cosolvent addition causing turbidity may be attributed to a local overconcentration of the cosolvent reducing FhuA and FhuA Δ1-159 solubility leading to precipitation of FhuA or FhuA Δ1-159. Supporting Material, Figure C, illustrates the precipitation and dissolution process and Fig. D (SDS-gel of precipitate) shows FhuA Δ1-159 which was precipitated. Precipitated FhuA Δ1-159 could be redissolved by supplementing a buffer solution containing oPOE (3 vol%) or urea (4 M) and incubating (2 h, see Supplementary Material, Table E). The latter indicates that the detergent oPOE plays a pivotal for the water solubility of FhuA or FhuA Δ1-159 in the presence of cosolvents (THF, EtOH, C/M).

Figure 1 shows the measured (dotted line) and fitted (solid line; CONTIN algorithm) CD-data. Recorded and fitted data are in agreement showing, for instance, a very similar shape, which is a good hint that the CONTIN algorithm can be used to analyze FhuA or FhuA Δ1-159 and likely other β-barrel shaped outer membrane proteins which are rich in β-sheet content. On the basis of the fitted data, the CONTIN algorithm “deconvolutes” the data, displaying the structural elements alpha-helix (α), beta-sheet (β) and random coil (r) of the respective samples (see Table 1).

Deconvolution of CD-data shows almost no changes in the amounts of each structural element; α-helix, β-sheet or random coil, upon addition of any concentration of organic cosolvent. We verified these results by repeating the experiments three times with different FhuA or FhuA Δ1-159 preparation. Interestingly, even in the presence of high concentrations of organic cosolvents, partial amounts of FhuA and FhuA Δ1-159 remained in solution and yielded CD-spectra which indicate that the overall β-barrel structure is still intact.

Reduced intensities of the FhuA or FhuA Δ1-159 structure from a distinctive β-sheet structure (Figure 1) could mainly be based on the aggregation of FhuA proteins in the presence of organic cosolvents and can be considered as a three-stage phenomenon: (1) The protein structure is very stable, only slight alterations of the original spectra can be seen; (2) Addition of a certain amount of organic cosolvent leads to aggregation of the protein (appearance of cloudiness) while after an incubation time of 75 minutes, FhuA dissolves again, forming a transparent solution. CD-spectra still show the typical minimum and maximum of a β-sheet peptide, but are less intense, compared to stage (1). It is assumed that in stage (2), the local concentration of organic cosolvent leads to partial protein aggregation. In stage (3), the typical β-sheet CD-spectra of FhuA or FhuA Δ1-159 is reduced in absolute values but retains its overall shape. The latter could be explained by the assumption that majority of the FhuA or FhuA Δ1-159 precipitate while a small fraction remains in solution despite of the presence of the organic cosolvent (see Supplementary Material, Figures C and D).

In the following, the three stage phenomenon will be explained on the basis of FhuA and FhuA Δ1-159 in the presence of THF (Figure 1 I.a/II.a). FhuA protein variants show structural integrity (stage 1) up to 25 vol% THF. The spectra exhibit the typical structure of a protein which is rich in β-sheet content. 40 vol% THF leads to protein aggregation, however FhuA proteins redissolve during an incubation period (75 min; stage 2). The addition of 50 vol% or 65 vol% THF leads to irreversible precipitation of FhuA or FhuA Δ1-159 during the 75 min incubation period (stage 3). Precipitate could be redissolved in a detergent (oPOE; 3 vol%) solution (see Supplementary Material, Figure E). Interestingly, CD-spectra still showed a β-structure which is based on the fact that not all FhuA or FhuA Δ1-159 molecules aggregated in presence of THF (see deconvolution of CD-spectra, Table 1).

### 2.2. Secondary Structure Analysis

In order to determine the percentage of each structural element from a CD-spectrum, it is important to employ the right method for analysis. This is especially important for the analysis of membrane proteins in which for instance the origin and the number of spectra in the reference data sets are decisive for deconvolution [24]. For secondary structure analysis of FhuA, the methods, with their respective percentage of β-sheets, are listed below: Secondary structure assignment of FhuA wildtype (FhuA WT, PDB entry 2FCP) reveals about 49% β-sheet content [12], while DSSP [25] indicates 53% β-sheets. Deconvolution of FhuA WT CD-data by the Andrade algorithm yields a β-sheet content of 51% [23]. It is commonly known that determination of β-sheet content in β-barrel proteins varies for the same β-barrel protein, even if the same deconvolution algorithms are employed. For instance, a report on the deletion variant FhuA Δ1-159 analyzed with CONTIN [26] shows a β-sheet content in the range of 60–65% [27]. The latter β-sheet contents are in reasonably good agreement with our β-sheet content values in Table 1 (58–73%).

Variations in β-sheet contents depend often on sample preparation and protein “purity”. For instance, β-sheets contribute four times less to CD-absorbance measurements than α-helical structure, so that even small amounts of impurities can affect β-sheet content values [28]. In addition, β-strands have in general a higher structural flexibility (barrels, sheets, propellers, β-helices) [22].

CD was originally developed for water-soluble proteins. Therefore, analysis of highly hydrophobic β-barrel proteins with a detergent shell is challenging for the CD-method. Studies on FhuA [27] and OmpA (outer membrane protein A) [29] illustrated that depending on the detergent-like environment and its concentration around the protein (in this case either defined by polymer, *n*-octyl-polyoxyethylene or different lipids), wavelength shifts can occur (e.g., blue shift in presence of polymer [27]). In addition, Chen and Wallace (1997) reported that non-aqueous solvents shift spectral peak positions in CD-spectra. Depending on the nature of the solvent, shifts can be either red-shifts (apolar solvents) or blue-shifts (polar solvents) [24]. The shift of the maxima at round 195 nm could be caused by the cosolvent effect reported by Chen and Wallace (1997).

CD-data analysis gives good clues on whether FhuA or FhuA Δ1-159 are correctly folded and stable in the presence of cosolvents besides maxima shifts caused by cosolvents, detergent effects on CD-spectra and general challenges such as selection of a suitable deconvolution algorithm and high purity of FhuA or FhuA Δ1-159.

FhuA is more stable than the cork-domain lacking variant FhuA Δ1-159. We assume that this is the result of more than 60 hydrogen bonds and nine salt bridges between the protein interior and the cork domain [16]. Bonhivers *et al*. (2001) performed stability studies of FhuA and FhuA Δ021-128, the latter one lacking most parts of the cork domain, and concluded that the wildtype is thermally more stable (FhuA: *T*_m1_ = 64 °C (cork domain unfolding) and *T*_m2_ = 74.4 °C (barrel unfolding)), more resistant to trypsin proteolysis and more stable to denaturing agents. These trends correspond well to our findings in respect of higher organic cosolvent resistance of the wildtype FhuA.

### 2.3. Solvent Effects

The structural integrity of proteins is determined by interactions within the protein and its environment. Protein molecules in solution are surrounded by a hydration shell in which water molecules are mainly attached to the protein surface by hydrogen bonds. Structural loss or denaturation of proteins by organic cosolvents is based on disturbance of this hydration shell [30] or disruption of the hydrophobic core.

Due to their high affinity to water, hydrophilic solvents will most likely strip the water from the protein. The hydrophilicity is directly proportional to the dielectric constant *ɛ* and the donor/acceptor characteristics of the solvent. In some cases, the water-protein interactions are so tight that hydrophilic solvents have difficulties in striping water away [31]. MeOH and EtOH (*ɛ* = 33 and 30) are able to accept and to donate hydrogen bonds to water. They can harm the hydrophilic parts of proteins. THF (*ɛ* = 7.5) is exclusively an H-bond acceptor while CHCl_3_ (*ɛ* = 4.8) is neither an acceptor nor a donor.

FhuA wildtype and its deletion mutant FhuA Δ1-159 are integral membrane proteins and therefore the hydrophilic parts of the protein are located in the upper and lower rim of the β-barrels FhuA or FhuA Δ1-159 and contribute to the solubility in water.

The middle section of the β-barrel itself is highly hydrophobic and covered with detergent. In nature, the FhuA membrane-embedded surface consists of 45.4% hydrophobic residues [4]. Therefore it was assumed that solvents with a high logP value (CHCl_3_ = 2.0, THF = 0.53, EtOH = −0.24, MeOH = −0.76) are less harmful for hydrophobic membrane proteins. For FhuA or FhuA Δ1-159 the expectation turned out to be valid for THF and EtOH. A C/M mixture should, due to its dielectric constants and the logP values of each component, affect FhuA or FhuA Δ1-159 in a comparable manner to THF or EtOH. However, why does the C/M mixture directly lead to protein aggregation? In the case of FhuA, the hydrophobic part of the purified FhuA or FhuA Δ1-159 is surrounded by the detergent oPOE which assists in FhuA or FhuA Δ1-159 solubility. In subsequent control experiments we investigated whether oPOE solubility depends on cosolvent concentrations. THF and EtOH can be mixed with 3 vol% oPOE without showing changes in the sample turbidity (see Supplementary Material, Table F and Figure G). The addition of C/M to 3 vol% oPOE caused an immediate change from a clear solution to a cloudy one that became clear over time. However, in most cases, “lipid-like” droplets remained visible after incubation. In conclusion, we do not expect that the organic solvent mixture C/M itself leads to protein aggregation/precipitation. Rather, FhuA or FhuA Δ1-159 precipitation is governed by interactions between C/M and oPOE which reduces FhuA or FhuA Δ1-159 solubility.

In the subsequent paragraph, interactions of THF, EtOH, and C/M on FhuA or FhuA Δ1-159 will be discussed on a molecular level. THF leads to H-bond formations between THF and water, forming a water shell around the THF molecules. As more THF is supplemented, competition of THF *O*-atoms for H-bond formation increases [32]. Our study suggests that this leads to disruption of bulk water of FhuA or FhuA Δ1-159, causing a minor alteration of the secondary structure (Figure 1). The water interacting with FhuA or FhuA Δ1-159 is not disrupted because most THF gets “caged” by water. It is known that until 0.2–0.4 mole fraction (53–73%), *O*-atoms of THF are not competing efficiently as hydrogen bond acceptors compared to water, preferring THF-THF interactions [33]. Increasing the THF (0.4–0.7 mole fraction; 73–90%) would most probably cause complete replacement of H-bonds between water molecules by H-bonds between THF molecules [32], leading to competition between the water surrounding the FhuA or FhuA Δ1-159.

Small amounts of EtOH show more perturbation of the protein structure in comparison to THF. Water molecules form a distorted cage around the EtOH molecule (located mainly around the methyl group) [34]. Higher EtOH concentrations perturb the FhuA or FhuA Δ1-159 water because of hydrogen bond competition with the hydroxyl group of EtOH and van der Waals interactions with the alkyl chain. Perturbation of the water comes with rearrangement of the detergent, covering FhuA or FhuA Δ1-159. The latter might result in decreased solubility and aggregation of FhuA or FhuA Δ1-159.

FhuA WT and FhuA Δ1-159 were both least stable in mixtures of C/M. Chloroform cannot act as a hydrogen bond acceptor and because of the chloride’s van der Waals dimension, hydrogen-bond donor characteristics are very limited. It is highly likely that the reasons for FhuA or FhuA Δ1-159 aggregation lie in the interaction of the organic cosolvents with the detergent oPOE (see Supplementary Material, Table F and Figure G) which covers the hydrophobic middle part of FhuA or FhuA Δ1-159. As a result MeOH might, due to the interactions between CHCl_3_ and oPOE, obtain easier access to water molecules and the H-bond network of FhuA or FhuA Δ1-159, which might promote FhuA or FhuA Δ1-159 aggregation and finally precipitation.

## 3. Experimental Section

All chemicals were of analytical grade quality or higher, purchased from Applichem (Darmstadt, Germany) and Sigma-Aldrich Chemie (Taufkirchen, Germany). The detergent *n*-octyl-oligo-oxyethylene (oPOE) was purchased from Enzo Life Sciences (Lörrach, Germany). Protein concentrations were determined using the bicinchoninic acid assay (BCA-assay) from Pierce Chemical Co (Rockford, USA). The organic cosolvents were acquired from Sigma-Aldrich (THF, M) and Applichem (EtOH, C). All experiments were carried out using Eppendorf Research^®^ (0.5–10 μL, 10–100 μL, 100–1000 μL) pipettes (Hamburg, Germany) with tips (S1111-4000, S1112-1020-c, S1111-0006-c) from Star Lab (Ahrensburg, Germany).

### 3.1. Strains

*FhuA* was provided on the pHK 763 vector by Prof. Volkmar Braun [35] and cloned into pPR–IBA1 (isopropyl-β-d-thiogalactopyranoside (IPTG) induced), using an *Eco*RI restriction site at the 5′-end and an *Xho*I restriction site at the 3′-end. FhuA and the FhuA Δ1-159 deletion variant contain as previously described a signal sequence to the outer *E. coli* membrane [36].

### 3.2. Expression and Extraction of FhuA and FhuA Δ1-159

The FhuA plasmids were transformed into the expression host *E. coli* B^E^ strain BL 21 (DE3) omp8 (F^–^ *hsdS_B_* (r_B_– m_B_–) *gal ompT dcm* (DE3) *ΔlamB ompF::*Tn5 *ΔompA ΔompC*) [37] and expressed in the Biostat ED fermenter from Sartorius (Göttingen, Germany), with a working volume of 10 L (tryptone-yeast extract media (1% tryptone, 0.5% yeast extract, 0.5% NaCl), 37 °C, 150 rpm, six-flate disc-turbine, sparged with air). The protocol for expression and extraction of the FhuA variants was carried out as described by Nallani *et al*. [36], with slight modifications: inoculation volume was calculated to reach OD_600_ of 0.1 in the main culture. After induction with IPTG, cells had grown further at 37 °C until OD_600_ reached 2.5. The culture broth was harvested by centrifuging (3220 g, 20 min, 4 °C, Eppendorf 5810 D, Hamburg, Germany). Pellets were kept at –20 °C until proteins were extracted. All buffers are potassium phosphate (P*i*) based (100 mM). After resuspension in lysis buffer (100 mM P*i*, pH 8, 5 mM MgCl_2_, 0.1 mM CaCl_2_), cells were disrupted by a high-pressure homogenizer (3 × 1800 bar pressure, Emulsifelx-C3 Homogenizer, Avestin, Ottawa, Canada). For incubation, an extraction buffer (100 mM P*i*, pH 8, 5 mM MgCl_2_, 0.1 mM CaCl_2_, 2% Triton-X 100) was supplemented. Outer membrane fractions were isolated by centrifuging (38,500 g, 45 min, 4 °C, Thermo Scientific, Sorvall RC-6 Plus, Rotor F–21S–8X50Y, Hamburg, Germany) and washed three times with P*i–*buffer (100 mM, pH 8.0). The two subsequent ultracentrifuge steps were performed in a Beckmann Coulter Optima TM L-60 Ultracentrifuge, rotor 60-Ti (170,000 g), using 100 mM P*i*, pH 8, 1 mM EDTA and either 0.1 vol% or 3 vol% oPOE.

### 3.3. Purification and Concentrating of FhuA and FhuA Δ1-159

Protein impurities were removed by gel-filtration (Sephadex G25-fine, GE Healthcare, München, Germany). FhuA Δ1-159 was concentrated (1800 g, 40 min, 4 °C, Eppendorf 5810 R, Hamburg, Germany) using a 10 kDa MWCO filter unit (Vivaspin 15, Sartorius, Göttingen, Germany).

### 3.4. Determination of Protein Purity and Concentration

Protein purity was determined by 10% acrylamide gels with 0.1% SDS running in a Biorad Mini-PROTEAN system (Hercules, CA, USA). For visualization of the bands, coomassie staining was used [38]. Protein concentrations were determined by the BCA-assay (Thermo Scientific, Rockford, USA). The BCA-assay quantifies the total protein amount; the IMAGE-J (Image Processing and Analysis in Java, Version 1.41) program was used to determine the amount of purified FhuA (1300 ng/μL) and FhuA Δ1-159 (600 ng/μL). Figure H in the Supporting Material shows the purified FhuA and FhuA Δ1-159 used in CD measurements.

### 3.5. Sample Preparation for CD Spectroscopy

The total sample volume was 150 μL in each measurement. 180 ng/μL FhuA (leading to 20.7 μL) or FhuA Δ1-159 (leading to 45 μL) were pipetted into a small glass tube (S 4-W, transparent, Nr. 300095–100, 4 mL, 44.5 × 14.5 mm, Chromatographie Service GmbH, Langerwehe, Germany), standing on a magnetic stirrer (stirring magnet: Nr. 001.106, 6 × 3 mm, Cowie, Middlesbrough, United Kingdom, stirring unit: Mot 2.5, Ika Werke RT10, Ika Werke, Staufen, Germany) with either 31.8 μL or 7.5 μL oPOE-based buffer (100 mM P*i*, pH 8, 1 mM EDTA, 3 vol% oPOE). The protein in the oPOE-buffer always displayed 35 vol% of the total sample solution. The remaining 65 vol% of the sample were prepared by the respective amounts of buffer (100 mM P*i*, pH 8, 1 mM EDTA) and organic solvents (THF, EtOH or C/M (1:1 v/v), 0%, 1%, 10%, 25%, 40%, 50%, 65% (v/v). Both the buffer and the organic solvent were slowly added by pipetting to the protein sample. Stirring was carried out for 75 min (in the subsequent text termed as incubation time). Each sample (150 μL) was carefully transferred with an Eppendorf pipette onto a Hellma^®^ SUPRASIL cuvette (Hellma GmbH & Co. KG, Müllheim, Germany) with a pathlength of 0.5 mm. Sample analysis was performed at room temperature with Olis SDM 17 CD (Olis, Bogart, USA).

### 3.6. Secondary Structure Determination of FhuA Variants by CD Spectroscopy

For each sample, five scans from 195 to 240 nm were recorded and averaged. 195 nm was selected as a benchmarking wavelength since organic solvents (like THF) have an increased absorbance below 195 nm leading to high noise/signal ratios. The latter interferes with deconvolution of recorded CD-spectra. The bandwidth was adjusted to 2 nm, the step width to 1 nm. As baseline, buffer (100 mM P*i*, pH 8, 1 mM EDTA, 1.05 vol% oPOE) without FhuA was employed in all experiments since the baseline in presence of cosolvent did not deviate significantly from the baseline of the buffer without cosolvent (see Supplementary Material, Figure A; example THF). In addition, deconvolution confirmed that the THF-baseline or oPOE-baseline can be used as reference for analyzing CD since both yield comparable β-sheet contents (see Supplementary Material, Table B). CD-spectra were smoothed by the Savitzky-Golay filter (Olis Global Works software package), and results were given as milli degrees and converted into mean residue ellipticity. To quantify the extent of changes in the secondary structure of FhuA and FhuA Δ1-159, the CONTIN algorithm [26], originally developed by Provencher and Glockner [39], was used. The algorithm is implemented in the Dichroprot software [40] and includes a spectra database of known secondary peptide-structures in order to predict the content of α-helix, β-sheet and random coil in the respective sample.

## 4. Conclusions

FhuA and FhuA Δ1-159 showed a structural integrity in up to 40 vol% THF and 10 vol% EtOH. In C/M mixtures, FhuA aggregates, starting from 10 vol% of organic cosolvent, while the deletion variant already indicates strong aggregation above 1 vol% C/M. Independent of the structural integrity of FhuA or FhuA Δ1-159, deconvolution of CD-spectra showed that even at high concentrations of organic cosolvent, FhuA or FhuA Δ1-159 did not greatly alter the examined structure. Furthermore, the cork domain improves the resistance of FhuA in organic cosolvents.

Our study showed that it is possible to investigate structural integrity of the β-barrel protein FhuA in organic cosolvents by CD-spectra analysis. We gained first insights of cosolvent effects on stability and solubility of FhuA or FhuA Δ1-159. The developed protocol opens opportunities to further study interactions of FhuA and other β-barrel proteins in non-natural environments. Polymersomes and artificial membranes offer novel applications for engineered membrane channel proteins such as hosts for hybrid catalysts or as filters in membranes or medical applications.

## Supplementary Information



## Figures and Tables

**Table 1:** CD-spectra of FhuA and FhuA Δ1-159 in the presence of varied amounts of the organic cosolvents THF, EtOH or C/M. CD-spectra were recorded in milli degrees and converted into mean residue ellipticity and fitted with the CONTIN algorithm implemented in the Dichroprot software, determining the amount of α-helix, β-sheet and random coil content of the respective samples.

	Secondary structure in %-age					
	
Sample	FhuA WT			FhuA Δ1-159		

	Helix	Sheet	Random coil	Helix	Sheet	Random coil
Standard	4	61	34	3	58	39
1 vol% THF	3	64	33	3	64	33
10 vol% THF	1	66	33	1	66	33
25 vol% THF	0	73	26	0	73	26
40 vol% THF	0	60	40	0	60	40
50 vol% THF	0	65	35	0	65	35
65 vol% THF	0	67	33	0	67	33
1 vol% EtOH	6	63	31	0	63	37
10 vol% EtOH	8	73	19	0	63	37
40 vol% EtOH	0	68	32	0	70	30
1 vol% C/M	8	66	26	0	69	31
10 vol% C/M	3	62	35	0	68	32
25 vol% C/M	0	67	33	0	67	33

## Abbreviations

CD: circular dichroism
THF: tetrahydrofuran
EtOH: ethanol
C/M: chloroform/ methanol
FhuA protein: ferric hydroxamate uptake protein component A
FhuA Δ1-159: FhuA deletion variant
oPOE: *n*-octyl-oligo-oxyethylene
BCA-assay: bicinchoninic acid assay
IPTG: isopropyl-β-d-thiogalactopyranoside
P*i*: potassium phosphate
EDTA: ethylenediaminetetraacetic acid
MWCO: molecular weight cut off
SDS: sodiumdodecylsulfate
α: alpha-helical structure
β: beta-sheet structure
r: random-coil structure
FhuA WT: FhuA wildtype
O-atom: oxygen-atom
H-bond: hydrogen-bond
