# Supplementary material for: First Insights on Organic Cosolvent Effects on FhuA Wildtype and FhuA Δ1-159
Source: Int J Mol Sci. 2012 Feb 22;13(2):2459–71. doi: 10.3390/ijms13022459 (PMC3292033; doi:10.3390/ijms13022459)
Supplement: Supplementary file 1 [file ijms-13-02459-s001.pdf]

Supplementary Information

1. CD-spectra of WT FhuA Protein in Presence of THF with Baselines Based on Buffer or Organic Cosolvent (o.s.)

Figure A. CD-spectra of WT FhuA protein in presence of varied amounts of THF (1–50 vol%) were recorded. Afterwards, either the baseline based on the buffer oPOE (1.05 vol%) (buffer) or the baseline based on the respective amount of the organic cosolvent THF (o.s.) were subtracted.

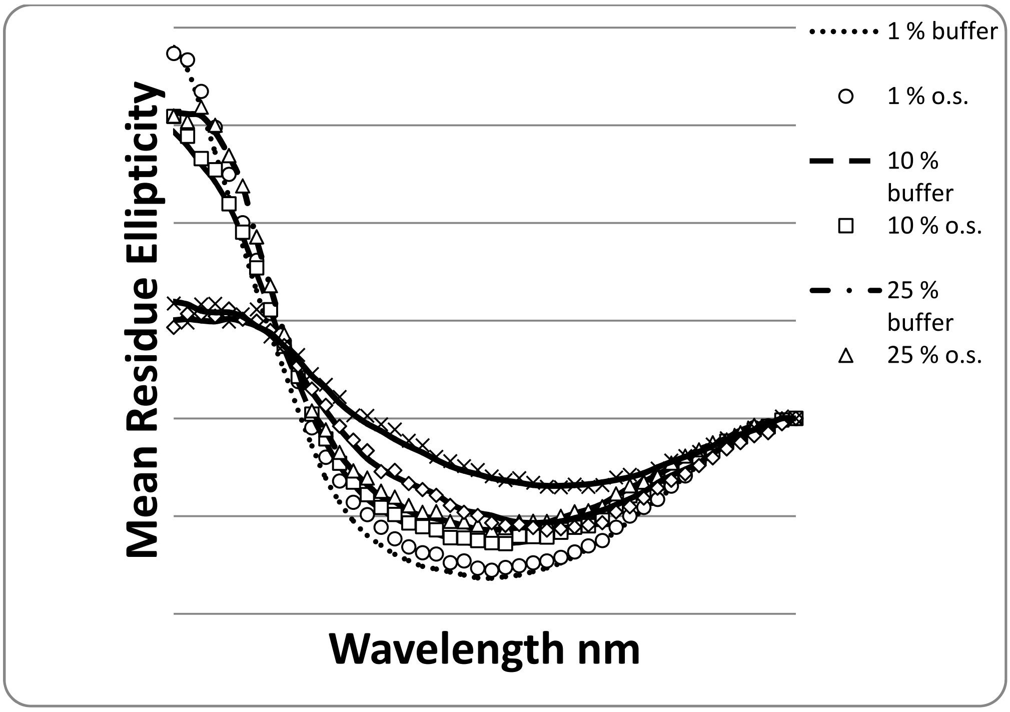

Only neglectable differences can be seen in the slope of the two spectra. In order to see the impact of the two different baselines on the amount of secondary structure found in the proteins, deconvolution of the recorded spectra was carried out (see next part).

2. Deconvolution of FhuA WT CD Spectra (THF) with Baselines Based of Buffer or Organic Cosolvent

Table B. CD spectra of FhuA WT in presence of different concentration of THF were either normalized by subtraction of buffer baselines or buffer/cosolvent baselines. Data were deconvoluted (CONTIN algorithm, implemented in the Dichroprot software) to obtain the percentage of the structural elements (α-helix, β-sheet and random coil).

Sample	FhuA WT buffer baseline			FhuA WT organic cosolvent baseline		
	Helix	Sheet	Random coil	Helix	Sheet	Random coil
1%	3	64	33	3	67	30
10%	1	66	33	1	65	34
25%	0	73	26	0	74	26
40%	0	60	40	1	62	37
50%	0	65	35	0	61	39

Deconvolution showed deviations of ≤15 %, which are within standard error rates and which can be attributed to difference in sample preparation/purity and measuring conditions.

The run of the curves (Figure A) and the deconvolution of the data (Table B) revealed in both cases a neglectable dependency on baselines chosen for shape of recorded CD-spectra and deconvolution. Therefore the buffer baseline was chosen as baseline for all measurements.

3. Addition of Cosolvents to FhuA Δ1-159 Leads to FhuA Precipitation.

Figure C. Preparation of FhuA Δ1-159 samples for CD analysis. The purified protein was transparent in solution (left, stage 1), addition of 10 vol% C/M resulted in an intransparent solution which cleared up after 75 min incubation time (2nd left, stage 2). Addition of 25 vol% C/M (3rd left, stage 3) resulted in an irreversible precipitation.

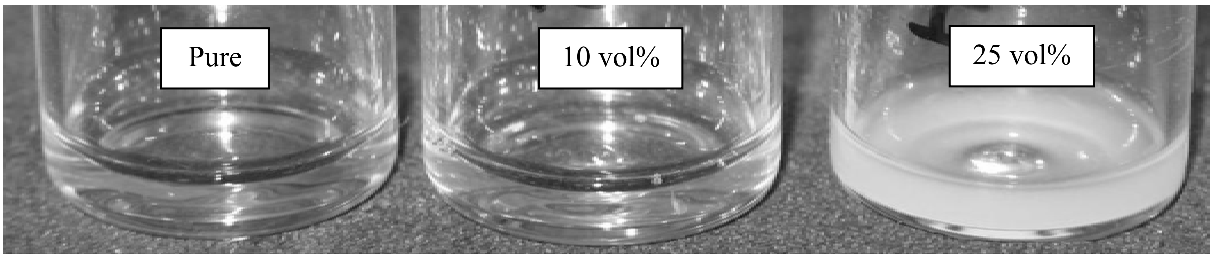

4. SDS-gel Proves that FhuA Δ1-159 (61.5 kDa) Precipitates

Figure D. FhuA Δ1-159 in solution and the precipitate (upon cosolvent addition) was centrifuged and loaded on SDS-gel. Staining with coomassie blue proves that the precipitate contains FhuA Δ1-159 (Figure C, 25 vol% C/M). M = Marker, 1 = FhuA Δ1-159 in solution, 2 = FhuA Δ1-159 particles after addition of C/M.

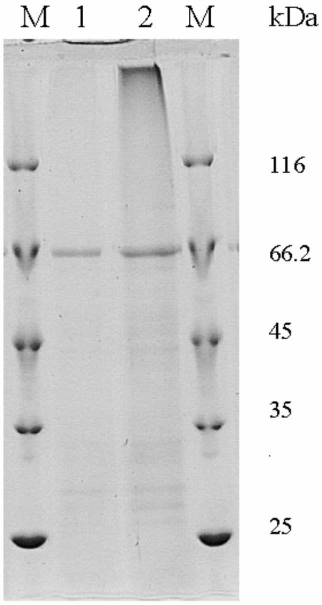

5. Dissolving of Precipitate with Detergent oPOE and Urea

Table E. FhuA Δ1-159 was precipitated by addition of 65 vol% THF or 40 vol% C/M, respectively and centrifuged (10 min; 10000 g). The supernatant was removed carefully by pipetting and either 1 mL 3 vol% oPOE buffer or 4 M urea (P*i*, 100 mM) were added to the precipitate. After 2 h shaking (RT), the absorbance at 600 nm (Sunrise, Tecan, Männedorf, Switzerland) was recorded.

	65 vol% THF	40 vol% C/M
3 vol% oPOE	0.037	0.048
4 M urea	0.042	0.055

FhuA Δ1-159 dissolved in both buffers upon oPOE or urea addition. The solutions cleared up nearly completely and the absorbance values are close to the absorbance of the buffer itself (see Table F). The ability of FhuA Δ1-159 to dissolve in detergent indicates the crucial role of the detergent for the solubility of FhuA Δ1-159.

6. Interaction of Organic Cosolvents with Detergent oPOE

Table F. Baselines (the respective amount of organic cosolvent in 1.05 vol% oPOE) were prepared and absorbance was measured at 600 nm (Sunrise, Tecan, Männedorf, Switzerland) directly after preparation (t = 0) and after an incubation time of 75 min (t = 75). Note that the data with grey background color were not used for this study but are shown to provide a full picture.

	% / t in min	0	1	10	25	40	50	65
THF	0	0.030	0.033	0.029	0.030	0.035	0.030	0.034
	75	0.031	0.031	0.033	0.032	0.035	0.036	0.033
EtOH	0	0.031	0.032	0.031	0.031	0.041	0.034	0.106
	75	0.031	0.032	0.032	0.033	0.056	0.038	0.035
C/M	0	0.033	0.941	0.742	0.418	0.413	0.430	0.111
	75	0.033	0.043	0.078	0.214	0.070	0.084	0.070

The data illustrate that the combination of THF with the detergent oPOE had no effect on the turbidity of the samples. After incubation time, the combination of 40 vol% EtOH slightly contributes to the turbidity (Δ 0.025). Similar results were obtained for the cosolvent mixture C/M below 25 vol%. Appearance of precipitation is due to the detergent oPOE (Figure G).

Figure G. Glass-microtiterplate showing oPOE precipitation in presence of varied amounts of organic cosolvents. Picture was taken directly after addition of different concentration of the organic cosolvents THF (D), EtOH (E) and C/M (F) (1 = 0 % o.s., 2 = 1 % o.s., 3 = 10 % o.s., 4 = 25 % o.s., 5 = 40 % o.s., 6 = 50 % o.s., 7 = 65 % o.s.).

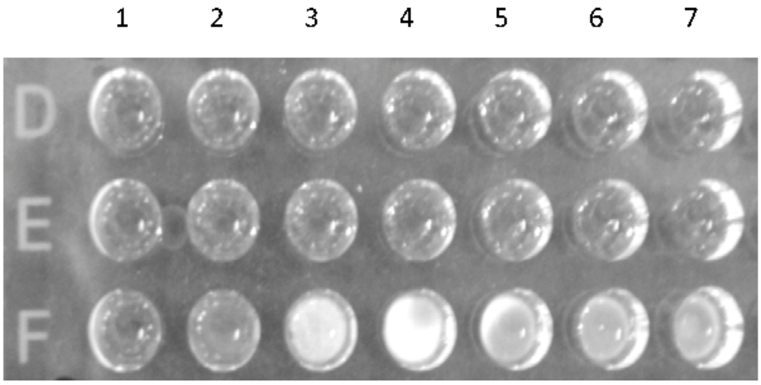

© 2012 by the authors; licensee MDPI, Basel, Switzerland. This article is an open access article distributed under the terms and conditions of the Creative Commons Attribution license (http://creativecommons.org/licenses/by/3.0/).
